# Skin Diseases and Syphilis

**Published:** 1893-08

**Authors:** 


### SKIN DISEASES AND SYPHILIS.

EDITED BY DR. M. B. HUTCHINS.

*Monatshefte für Prakt. Derm.*, XVI. No. 11, quotes, from *Semaine Medicale,* Hale as having used pilocarpine injections in two children, of seven and nine months respectively, affected with erysipelas. Injections sufficient to produce sweating. Original high temperature and brain symptoms improved in twenty to thirty hours.

### ANTISEPTIC TREATMENT OF FURUNCLE AND CARBUNCLE.

Burlureaux recommends baths or dressings with carbolic solution, or a five per cent. creolin solution in the beginning, also spraying with one of these and interstitial carbolic injections. After opening and the removal of the core or necrotic mass, packing the opening with equal parts of quick lime (!), carbonate of soda and alum.—*Monats. für Prak. Derm.*, XVI. No. 11.

### SYMMETRICAL OCCURRENCE OF CONNECTIVE TISSUE TUMORS.

*Deutsche Zeitschrifte für Chirurg.* Bd. xxxiii, p. 612, contains report of the case of a seaman thirty-two years old, very much run down through gonorrhœa, syphilis, malaria, "beri-beri," etc., showing on each elbow and over each trochanter major a small, knobby, irregular subcutaneous swelling. One removed from the right arm showed fibro-sarcomatous structure. Etiology uncertain.

### THE MICROBE OF SOFT CHANCRE.

*Monatshefte* also refers to an article by Rudolph Krefting upon this subject in *Ann. de Derm. et de Syph.,* discussing the question as to the identity of the bacillus found by Ducrey in chancroidal pus and that found by the writer in a virulent bubo with the strepto-bacillus found by Unna in sections of chancroidal tissue. He answers in the affirmative, attributing differences in the bacteria to the different conditions of growth in the different media. The duty of artificial cultivation and production of chancroid upon men or animals with the cultures yet remains.

---

### CONGENITAL TUMORS.

*Monats. für Prak. Derm.,* XVI. No. 11, quotes von Pott (*Munchen. Med. Wochenscrifte*) as follows: Congenitally vessel tumors are most frequent, as angioma, cavernoma, lymphectasies, lymphangioma (Hygroma Collicysticum). Lipomata are also often seen, in which one frequently finds calcareous deposits. In the genito-urinary system are found, besides teratoma and cystadenoma of testicle or ovary, frequently sarcoma of the kidney. Further, sarcoma of the optic nerve (glioma) may exist, also cases of cartilage or bone tumor.

---

### NATURE OF THE VERNIX CASEOSA.

Dr. Wallace Beatty, whose article in *The British Journal of Dermatology* upon the functions of the sweat and sebaceous glands was mentioned in these pages, has reported in that journal, July, 1893, having examined the vernix caseosa of the scalp of an infant dying a few minutes after birth. Sections of the skin were stained with osmic acid, blackening the fatty portions. Under the microscope the blackened fatty layer on the surface was found directly continuous with the blackened fat filling the hair follicles and sebaceous glands. Neither the lumen nor the walls of the sweat glands or ducts showed any blackening, proving that the vernix caseosa is derived from the sebaceous glands and not from the sweat glands as Unna held.

### MISTREATED SKIN DISEASES.

It is well, in investigating any skin trouble, to ascertain what applications have been used before you saw the eruption, as these may have modified the appearance in such manner as to mask the original symptoms. The most of the "homely remedies" may also have intensified the original trouble, and deserve to be classed as causative of much which you see.

An acute dermatitis recently seen by the writer had received liberal applications of *salt and water* and *sulphur and lard.* The intelligent reader can imagine the result.

---

### GONORRHŒAL IMPLICATION OF THE SEBACEOUS GLANDS OF THE PENIS. Dr. Touton (*Berliner Klinische Wochensch,* No. 57, 1892).

The author reports two cases in support of his previous paper (*Ueber folliculitis præputialis et paraurethralis gonorrhoica. Archiv. für Derm. und Syph., xxi.,* p. 15), and gives observations which go to show that the sebaceous glands of the penis may become affected in the gonorrhœal process, and make it probable that the greater part of the so-called paraurethral and preputial ducts are sebaceous glands altered by the disease.

It is further demonstrated that the gonorrhœal virus is capable, also, in this tissue, of causing exuberance and alteration in the epithelium; and finally, is shown the possibility of chronic gonorrhœal inflammation persisting here as well as inflammation which lasts after the disappearance of gonococci, or which may exist where they have been absent, depending upon other micro-organisms.—*Journal Genito-Urinary and Cutaneous Diseases, July,* 1893.

---

### TREATMENT OF BUBO. Spietschka (*Prager. Med. Wochens,* No. 34, 1892).

The writer advises early and complete removal of the diseased glands, or if suppuration has occurred a small incision and removal of detritus by means of a curette.

Welander has given the following formula with the view of producing abortion of bubo.

℞. Hydrag. benzoatis, gr. v.
Sodii chloridi, gr. jss.
Aq. destil., ℥ j.
M. S. m. XV to be injected.

This causes some pain and does not always succeed.

In twelve cases in which this was recently tried, a small incision being also made because of fluctuation, a cure was obtained on an average of twenty-eight days.

In Pick's clinic compresses moistened in acetate of aluminia are found less irritating to the skin and can be left on for two days. At times one injection of the one per cent. benzoate of mercury is not sufficient, and at others the method succeeds even after fluctuation is present. In sixty-two cases treated over thirty-seven per cent. were cured by the injections alone, while in sixteen cases a free opening and resort to the curette was found necessary later on.—*Journal Genito-Urinary and Cutaneous Diseases, July, 1893.*

